# Social Perception of Zoos and Aquariums: What We Know and How We Know It

**DOI:** 10.3390/ani14243671

**Published:** 2024-12-19

**Authors:** Ana Villarroya, Rafael Miranda, Andrea Pino-del-Carpio, María Casas

**Affiliations:** Instituto de Biodiversidad y Medioambiente (BIOMA), Universidad de Navarra, 31008 Pamplona, Navarra, Spain; avillarroya@unav.es (A.V.); andrea.pinodc@gmail.com (A.P.-d.-C.); mcasas.1@alumni.unav.es (M.C.)

**Keywords:** animal welfare, public perception, research gaps, visitor impact

## Abstract

Zoos and aquariums attract millions of visitors around the world. At the same time, the functions they fulfill and the way they do that have been subject to scrutiny from different fields of knowledge. The way societies perceive animals has evolved in recent decades and so has the social perception of institutions that work with them. This scoping review aims to gather studies from the last three decades that shade light on how current societies perceive zoos and aquariums. We selected papers describing quantitative studies published in English in peer-reviewed journals. We reviewed 71 works from different fields of knowledge. The proportion of female authors was noticeably higher than in general science. Most studies were carried out in culturally Western countries, a fact that constitutes a gap in knowledge from a global perspective. We also noticed an underrepresentation of aquariums and of children in the analyzed papers, and a need for more pre–post studies to ascertain the effect of a zoo or aquarium on its visitors. These conclusions point out interesting directions for future studies to help scientists, managers, and citizens design the future of these institutions.

## Simple Summary

People are increasingly concerned about the treatment and wellbeing of animals in zoos and aquariums, which has led to a big shift in how these places are seen by the public. For zoos and aquariums to keep up with these changing views, they need to understand how society thinks about them. Over the past 30 years, many studies have looked at how people feel about zoos and aquariums. This review seeks to compile recent research that offers insight into how contemporary societies view these establishments. One finding is that a higher number of female researchers have been involved in studies on zoos and aquariums compared to other areas of science, and previous studies have reported that women tended to be more empathetic towards animals and animal welfare than men. Most of the research has been conducted in Western countries, which creates a gap in our understanding from a global point of view. The studies also did not focus much on aquariums or children, and there is a need for more research that assesses changes in visitors’ perceptions of animal wellbeing and the role that zoos and aquariums play in species conservation that compares attitudes before and after their visit. These insights highlight important areas for future research that could help shape how zoos and aquariums evolve and improve.

## 1. Introduction

The way humans coexist with animals and with the environment they live in is a hot issue nowadays. Especially since the 1970s, how industrialized societies treat animals has increasingly been questioned, e.g., [1,2].

One of the most significant expressions of this phenomenon is the concern about animal rights and welfare. Albeit not new, these concepts have certainly gained popularity in the last few decades [3]. The evolution of national and international legislation and codes of practice on animal welfare in the previous century reflects this changing sensitivity [4,5,6,7]. Social movements advocating for these issues have spread, and the number of organizations promoting the animal cause has grown significantly [8]. The media also showcase this discussion, as it is easy to find news related to animal welfare and animal rights in mainstream media. For some analysis, see [9,10,11].

Food, clothing, research, and entertainment are some sectors that work with animals in industrialized countries, whose practices are under scrutiny in light of emerging ethical concerns towards animals. A growing core of academic publications discussing these procedures provides valuable insights into defining future directions for the industry [12,13]. However, in addition to theoretical approaches, industries need to know consumers’ perceptions about their practices to adapt to their demands. Several studies have adopted this focus regarding, among others, public perception of farm animal welfare [3,14,15], the use of animals in research [16,17], and the existence of zoos and aquariums [18,19,20]. The latter is the focus of this paper.

The first zoos appeared in the Chinese, Roman, and Egyptian civilizations about 4500 years ago [21]. They were private collections of animals with a double purpose: to show their owners’ power and wealth and to entertain their guests. This zoo concept remained more or less unaltered until the 1950s–1960s, when the first zoological societies were born. Those organizations promoted public zoos [22], which started carrying out conservation and research functions [23] in addition to entertainment. This started a structural and ideological transformation in zoos [24], greatly motivated by growing environmental awareness and moral concerns against animal captivity [25]. This stance has spread since the 1990s, and today, the very existence of zoos is still a controversial matter under debate [26,27,28]. As a result of this process, by the end of the 20th century and the beginning of the 21st, zoos and aquariums conceived themselves as facilities that address four functions: species conservation, research, environmental education, and entertainment [29]. However, balancing those roles proves difficult, as zoos heavily depend on the entrance fees of visitors mainly looking for entertainment [30].

Notwithstanding all the controversies, zoos attract millions of visitors each year. In 1992, Wilson reported that more people visited zoos and aquariums than attended professional athletic events in the USA and Canada [31]. In 2011, Gusset and Dick stated that zoos and aquariums get over 700 million visitors worldwide each year [32]. The public attending zoos comprises a diverse range of demographics and interests [33]. These data suggest that “zoos are in a unique position to provide environmental education and conservation education to large numbers of people” [34], especially for an urban population who seeks opportunities to connect with nature [35]. In a global crisis [36], such educational potential may be of utter importance. At the same time, keeping animals in zoos is controversial, even with educational or conservation goals. Some authors argue that there is insufficient evidence of zoos’ educational or conservation impact to justify such animal welfare costs [37,38,39]. As a result, zoos now need to demonstrate their effectiveness in these areas to account for their importance in the eyes of society [40,41].

In the last two decades, several academic publications have addressed social perceptions about zoos and aquariums, mainly focusing on a specific place (country or facility) or public. They all provide valuable insights into how current societies perceive zoos and aquariums. Given the growing relevance of this issue and the need for further studies, a review of the state of the art seems timely.

This scoping review aims to gather evidence on how social perception of zoos and aquariums is assessed. We pay attention to (1) bibliometric characteristics of published studies (who, when, and where such works are carried out) and (2) what evidence these papers give us (and what they do not) about how people perceive these institutions.

## 2. Materials and Methods

We performed a systematic search of academic publications following the PRISMA guidelines [42]. We used Web of Science and Google Scholar as search engines and databases and looked for citations in the selected references (backwards and forward snowballing). We executed the last search in October 2024. We did not restrict the knowledge area of our searches, as the studies we were looking for may come from diverse disciplines. We looked for the following keywords in the title, abstract, or keywords fields: zoo perception, aquarium perception, visitor perception, visitor interest, visitor motivation, tourist perception, animal welfare, and animal preference.

We only selected papers published in peer-reviewed journals in English but set no limitations regarding impact metrics or inclusion in top academic ranking systems. This criterion allowed us to include journals of restricted diffusion, which we deemed interesting for our study because they publish works from countries that often seem misrepresented in general scientific journals. We cross-checked the results with Beall’s list (https://beallslist.net/, accessed on 30 October 2024) to discard possible predatory journals. We included papers published within the last three decades (1993–2024) to ensure they were contemporary to the recent changes in sensitivity towards animals. Eligible studies need to focus on the social perception of zoos, aquariums, or their animals; describe experimental studies; and provide reliable results. We performed two-step screening using the Covidence software for peer analysis (https://www.covidence.org/, accessed on 30 October 2024), first assessing the title and abstract and then considering the whole text. Each reviewer independently voted whether to include or exclude each paper from the review. In case of consensus, the software automatically moved the paper to the following stage in the review. Conflicted results were resolved in face-to-face discussions. According to the PRISMA guidelines [42], when the reviewers disagreed on whether to include a publication, they discussed it personally according to previously exposed eligibility criteria (English language, non-predatory journals, focused on social perception, experimental studies, and reliable results).

Table 1 shows the types of data extracted from the selected papers. To standardize and streamline the process, the authors worked in parallel in Covidence using an extraction template.

## 3. Results and Discussion

### 3.1. How We Know About Zoo Perception: Characteristics of the Reviewed Studies

**Figure 1:**
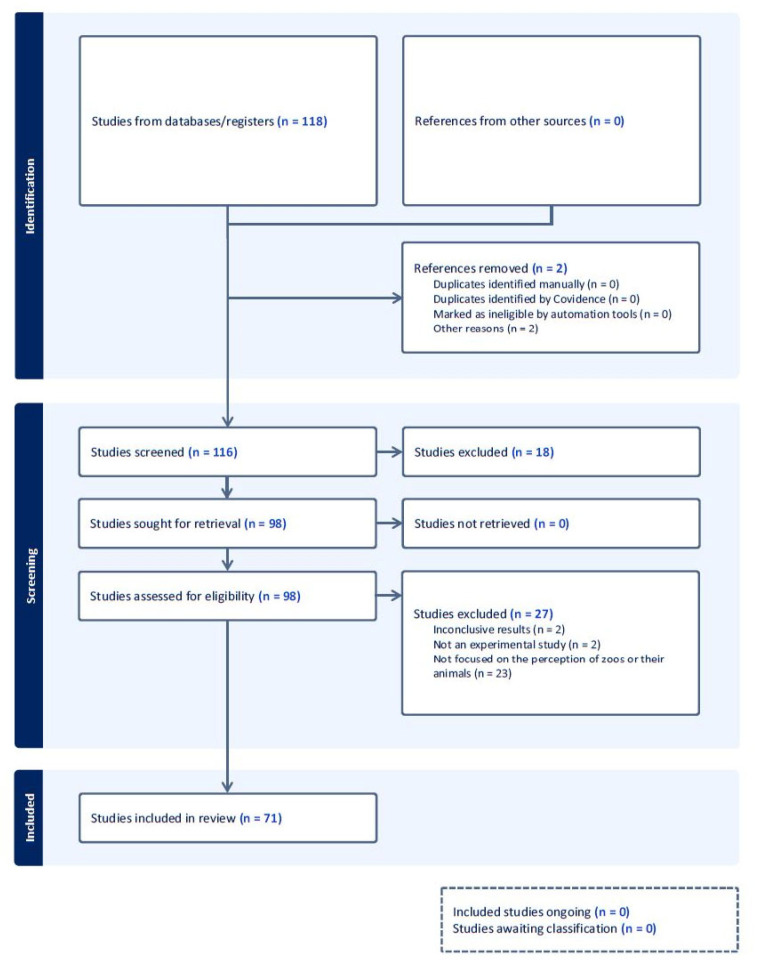
PRISMA flowchart summarizing the screening process [42].

The electronic searches finished on October 2024 and yielded 118 scientific papers. We discarded 47 papers for failing to meet one or more of the eligibility criteria, leaving 71 studies to be reviewed (see Figure 1). Table S1 shows a list of the reviewed papers.

The two most popular journals among the reviewed papers were *Zoo Biology* (12 articles) and *Anthrozoös* (10 papers). Both belong to the “Veterinary sciences” category in JCR, although *Zoo Biology* also falls within the category of “Zoology”, whereas *Anthrozoös* is also a sociological journal. Another 11 papers appear in journals in the JCR category “Hospitality, leisure, sport & tourism”.

Of 214 authors, 118 (55%) were female and 90 (42%) male, whereas six authors (3%) were classified as “other”. On average, the sex ratio was approximately 1:1 (52% females and 48% males). However, only 22 of the 71 reviewed papers had less than 50% female authors, and 13 had only female authorships. These results contrast with global data on women’s research output since, in general terms, women account for approximately 30% of fractionalized authorships [43], a gender gap that widened during the COVID-19 pandemic [44,45]. The proportion of female authors in the reviewed papers is also higher than historical data on specific fields such as veterinary medicine (32%) or sociology (30–41%) [46,47]. In this context, the result of 55% female authorships in our review is noticeable, as there is a greater feminine representation than we could expect at first. Although more exhaustive analyses would be necessary to ascertain the reasons for this gender proportion, previous studies have reported women to be more empathic towards animals and animal welfare than men [48,49].

**Figure 2:**
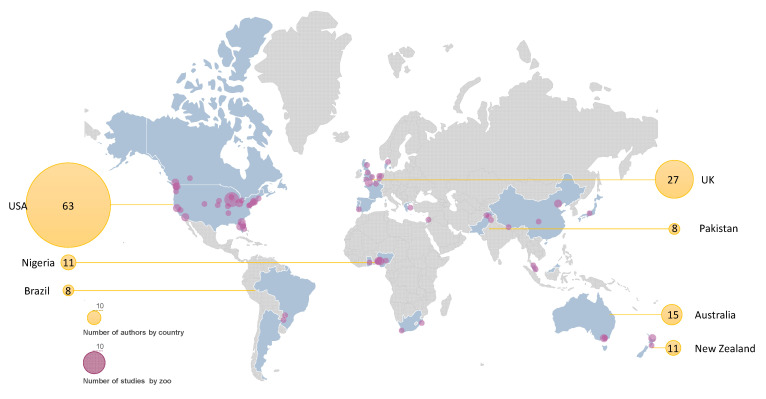
Countries that host authors’ affiliation institutions (in blue). Number of authors per country (in yellow) and the number of studies carried out in each zoo/aquarium (in purple).

Most authors’ affiliations corresponded to universities (72%). However, two of the most popular institutions (as for the number of times they appear as an author’s affiliation) were zoos: the Chicago Zoological Society–Brookfield Zoo and Lincoln Park Zoo. The country most often listed as authors’ affiliation was the USA (28 papers, 63 authors), followed by the UK, New Zealand, and Australia (Figure 2). These results partially align with the country ranking of publications (1996–2023) developed by the SCImago research group [50], which shows the USA as the country that publishes the highest number of scientific papers worldwide. The UK ranks third in the general assessment but second in the “Veterinary” category. However, New Zealand and Australia (3rd and 4th in our evaluation) ranked 28th and 11th, respectively, in that category (39th and 10th in the general assessment), also being among the regions that host the lowest annual number of visitors [32]. Perhaps the fact that many Australian zoos put a strong emphasis on their conservation role [51,52,53] might somehow spur higher scientific production rates in this area than expected. The least represented region in our review was South America, with only two countries (Argentina and Brazil) and three papers. There were only three African countries (Ghana, Nigeria, and South Africa) associated with seven papers. This result partially aligns with the total number of scientific publications (general and “Veterinary” category) reported by SCImago, where African and South American countries dominate the lower half of the country ranking. In our assessment, Asia has a wider variety regarding the number of countries cited as affiliations (seven countries) but with the same number of papers as Africa (seven). The absence of India and the low presence of China are noticeable, as these are two prolific countries regarding their number of publications in general and in the fields of veterinary sciences and conservation biology [31], and also host a high number of zoo visitors annually [50]. This result might stem from cultural differences in the importance that societies give to animal issues [54], which may deem the social perception of zoos not as relevant as other research topics in those countries.

This uneven distribution of publications by country—that Davey already pointed out in 2006 [55]—may represent a knowledge gap to address, as social perception is highly influenced by socio-cultural issues that vary among places. Our knowledge about how people perceive zoos and aquariums and their animals mainly comes from culturally Western, English-speaking countries, whose worldviews may differ greatly from other places or traditions. This is in line with what other authors who are researching animal perception have observed [48].

It should be noted that this review only considered English-language publications. This restriction could favor culturally Western countries and cause a sampling bias for English speakers and countries. However, more than 98% of scientific publications are written in English, despite the disadvantage for non-native English speakers [56]. Considering this percentage, this possible bias should be considered minor or irrelevant in a global scientific context.

Seventy-two institutions took part in the reviewed studies, although not all the papers disclosed the names of the participating zoos or aquariums (Figure 2). Chicago Zoological Society–Brookfield Zoo was the most cited entity, appearing in seven studies. Most studies cited one to three facilities, with some exceptions: Ballantyne and Packer [57] included fourteen zoos and aquaria, and Miller and collaborators [58] included nine entities. Two papers [59,60] described big-scale studies with 191 zoos and 486 aquaria, respectively.

Most papers describe research conducted in zoos (37 papers) or mixed facilities (meaning zoos that also host some aquatic animals, 19 studies). Eight studies did not disclose the facility where they carried out the study, and ten papers did not take data from such institutions (studies addressing the general population). Only five papers described research carried out in aquariums, leaving these institutions notably underrepresented compared to zoos.

The USA hosted the highest number of zoos and aquariums participating in social perception studies, with 30 disclosed institutions in 24 papers. Second was Nigeria, with seven institutions in five publications, followed by the UK, with five zoos participating in six studies.

**Figure 3:**
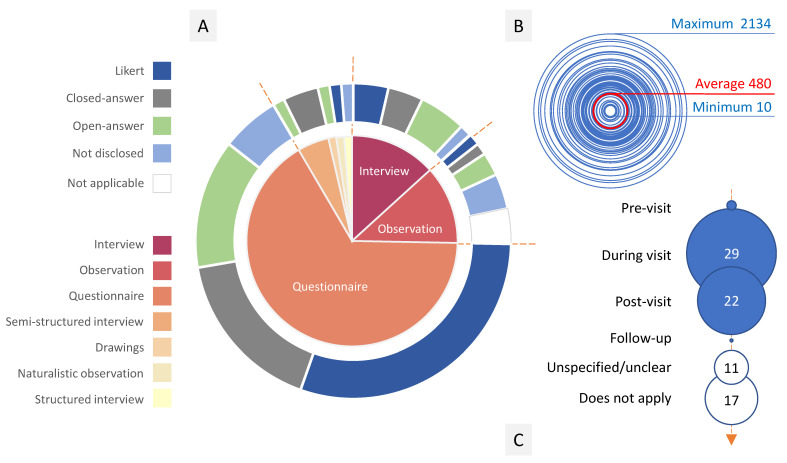
(**A**) Tools used to gather social perception in the reviewed studies, (**B**) sample size, and (**C**) number of studies that report collecting data at each point in time of a visit to the zoo/aquarium.

Most papers describe studies taking between one and four months long. The mean time gap between study completion and publication was three years. Most studies (98%) used questionnaires and interviews to gather public perceptions of zoos and their animals (Figure 3). Since interviews are usually based on a set of established questions—a questionnaire—to make them structured and help gather data more systematically, these two methods are closely related (or, in some papers, even considered as synonyms). Approximately one in every five studies used observation to gather data. Other data sources were children’s drawings, tourist reviews on a traveling website, focus groups, and autoethnography. Although not every paper using questionnaires disclosed their contents, most of those providing such information applied a combination of closed-answer and Likert-scale questions. These answers are quicker to fill in during surveys and streamline data analysis afterward, so it is no surprise they are popular instruments in social perception studies.

The sample size ranged from 10 to 2134 subjects, although the average size was 462 (Figure 3). Most studies addressed zoo visitors, but some aimed at the general population and zoo staff (Table S1). Many papers did not disclose the age of their respondents, but those providing such information tackled mainly adults. Six studies addressed families, and just one specifically tackled children. However, infants constitute a high percentage of visitors to zoos and aquariums [61]. Moreover, children are one of the main motivators for adults to visit these facilities. The lack of data about how they perceive these facilities and their animals constitutes a gap in knowledge that future studies should address.

Most studies tackling visitors gathered their data during visits or right after them (40), a strategy that allows them to obtain a snapshot of the public’s perception (Figure 3). Studies assessing how the zoo experience influences visitors must compare data taken at different moments. Four papers took data during and after the visit, and another two compared pre- and post-visit. One study [62] gathered data from visitors at four points in time: before the visit, during it, after its finishing, and two weeks after it. Studies comparing data from different points in time of the visit need more time and resources. In addition, they are less efficient in gathering data, since study subjects may be lost at each point in time. These facts probably explain why this kind of study is so scarce. However, this is the only way of assessing a zoo’s impact on its visitors, a challenge these facilities face now as they find their educative role highly questioned [63,64].

### 3.2. What We Know About Zoo Perception: Findings of the Reviewed Studies

As it seems to happen regarding many other phenomena, perception of zoos/aquariums and their animals may firstly depend on socio-demographic variables, such as gender and age [65]. For example, Muller and collaborators [66] report that women show greater empathy than men, whereas Alba and collaborators [67] stated that gender was important for predicting attitudes towards zoo animals. These statements align with previous studies reporting gender as a predictor of felt closeness or concern about animals, e.g., [3,68], although the influence of this single factor might be weaker than other determinants [69]. Some of the reviewed studies conclude that the perception of animals and zoos seems different in youths and adults [67,70]. However, as pointed out earlier, only one of the reviewed studies specifically addressed children [71].

Country of origin and cultural background influence how people perceive zoos and the animals they host [72,73]. For example, in a study by Gurusamy [74] about the perception of zoo elephants, Australian individuals focused on scientific value more often than Indian respondents, who tended to highlight religious and cultural attributes. In a study carried out with zoo staff [75], European interviewees highlighted the role of zoos in preserving biodiversity and safeguarding sustainable populations, whereas Chinese individuals focused on keeping animals safe and happy. The conceptual closeness between humans and animals can also shape how people perceive zoos. Muller and collaborators [66] reported that visitors who saw humans as not so similar to animals ranked entertainment as an important role of zoos. In contrast, visitors who reported greater similarity between humans and animals tended to point out conservation as one of the main roles of these facilities. However, animal perception is not static and may vary over time depending on experiences [76], education, and even information received through social media and the Internet [77].

In the reviewed studies, visitors and the public perceive education and recreation as the main functions of zoos and aquariums. This result partially aligns with the emphasis that many zoos give to entertainment on their websites [22]. Although research and conservation are also named, they are usually perceived as less preeminent than the other two, with some exceptions: see [18,20,23]. This is in tune with reported visitors’ motivations for attending zoos, mainly social–recreational and educational. Zoos’ ability to convey educational messages plays an important role in visitors’ perception of these facilities [57,78]. Several studies from different countries state that visitors identify zoos and aquariums as places where they spend family time while learning about animals, thus aligning recreational, learning, and social motivations [62,78,79]. Therefore, it is unsurprising that some studies state that people with children visit zoos more often than those who do not have kids [80].

Although the success of zoos’ educational work depends on several factors, a positive perception of zoos and their animals could improve visitors’ learning outcomes [39,81]. On the other hand, knowing how the public perceives certain activities may help refine educational tools to convey conservation messages more effectively. Direct observation of zoo animals is a positive experience that elicits visitors’ interest in animals and animal conservation [58,82], constituting a unique educational tool that combines leisure and learning [83]. In addition, interpretation by the staff and shows or presentations positively influence guest perceptions [84,85]. Regarding educational signs, visitors expect them to contain scientific information about the animal and its conservation [23,57]. However, some authors report that a small percentage of the public stops and reads them [83].

The characteristics of the animals’ exhibits greatly influence visitors’ perception. All the reviewed studies that tackle this issue agree that the public—even non-visitors—prefers naturalistic settings, which are also beneficial to animals [57,80,86]. Naturalness seems to have a greater effect on visitor perception than other components of the animal’s zoo environment, like enrichment materials. There is no consensus as to what degree these behavioral enrichment items may affect people’s perception of zoo animals, as some studies report no effect [87,88] while others state they may have an impact [89,90]. In addition, the presence of artificial environmental enrichment devices does not seem to alter the perceived naturalness of an exhibit [70,91]. While some studies report that visual barriers that conceal animals may not have a detrimental effect on visitors’ attitudes or experience [92], structures that make animals look restrained (such as bars) negatively alter the perceptions of animals in zoos [81].

Primates and big mammals (such as lions, tigers, or elephants) are the most popular zoo animals for visitors [29,65,73,86,93,94,95] and non-visitors in the reviewed studies [96]. Birds, reptiles (especially snakes), amphibians, fish, and invertebrates are the groups that people pay the least attention to or even perceive most negatively. These results are in tune with literature results that identify large exotic terrestrial animals as the most charismatic species in general [97] and within the context of zoos [98], at least in Western contexts. In our review, the authors identify several traits that make a zoo animal popular. Big, active animals, as well as babies, are attractive to visitors [29,72,92,94,96,99,100]. Animals with a friendly nature and those considered aggressive seem popular among visitors [65]. Conservation status and phylogenetic closeness to humans also seem to elicit positive reactions in the public [96,101,102], as reported in other studies [97].

Twenty-five out of the seventy-one reviewed studies address animal welfare in some way. Of them, 16 were published in the last four years. This fact reflects the growing interest in this topic that has taken place in recent times in different disciplines and environments, probably due to an increasing questioning of animals’ moral status and its implications for human–animal relations. However, most studies addressing the perception of animal welfare tackled big animals in zoos; we found little evidence regarding aquaria and smaller animals, a gap in knowledge that McGaw and collaborators pointed out in their 2023 study [60].

Visitors identify active behaviors as a sign of good animal welfare [90,92,103], whereas they associate stereotypical activities with poor care levels, decreasing their interest in zoos [19]. Naturalistic settings and big enclosures may make visitors perceive better animal welfare [58,103], while behavioral enrichment does not seem to have any effect [87]. This is in line with the definition of welfare that Ogle [84] reported in visitors, who associated that concept with the naturalness or wildness of animals. Visitor characteristics such as preferred animals, previous experiences, education, and social–cultural background can influence perceptions of animal welfare [104,105,106]. In addition, the relationship between an animal and its caretaker, or with other animals, can significantly influence visitors’ perception of animal welfare [107]. Emotional reactions and perception of animal welfare seem closely related [108]: positive emotional experiences and emotional connection with animals elicit perceptions of higher animal welfare [58,109]. This process seems to be bidirectional, as Sherman [110] reported that visitors who perceived giraffes to be in a better state of welfare had more positive emotional reactions. Zoo animals elicit emotions in visitors, both positive (excitement, surprise, curiosity, and joy) and negative (sadness and worry), influencing empathy development towards animals in young visitors [75]. Both kinds play a role in public engagement with animals, as positive emotions deliver an enjoyable experience while negative ones may help construct meaning [111].

Scoping reviews such as this have intrinsic limitations, and so do the authors who have performed them. We acknowledge that we may have unintentionally missed some relevant studies in our search and that the discussion of our results may benefit from complementary views from other fields of study. That said, we think that our work offers a relevant overview of the state of the art and allows for envisioning future directions for research.

## 4. Conclusions

How people perceive zoos/aquariums and their animals is a growingly interesting research subject in the present socio-cultural context. The reviewed studies add up to a thought-provoking body of knowledge that must be further developed in some aspects.

The data showed that aquaria are underrepresented compared to zoos in these studies, as most papers mainly focus on the latter. This deficiency constitutes a knowledge gap, although it is no surprise since aquariums usually host no charismatic species (which are commonly large, terrestrial vertebrates). Ultimately, humans’ low affinity towards fish, amphibians, reptiles, and invertebrates may indirectly influence research interests within this field of knowledge.

Our study revealed that gender may be a relevant variable regarding research interests and visitors’ perceptions. The proportion of female authors within the reviewed papers was noticeably higher than in general science, and the results of the perception studies reported women to be more empathic towards zoo animals than men. This is in tune with similar conclusions from other studies within the field of human–animal interaction, although further research is necessary to ascertain such a relation in detail. Besides gender, age also mediates the way we perceive animals. However, even if children represent a significant proportion of zoo and aquarium visitors, we found very few studies that addressed this age group. If these facilities want to fulfill their functions in the best possible way, ascertaining children’s experience and valuation seems necessary.

Social perception is highly dependent on the cultural context, making it difficult to extrapolate conclusions from one region to another. However, our research found an uneven geographical distribution of perception studies since these were more numerous in culturally Western countries than in other areas. This fact operates two ways: it represents a gap in knowledge, as there is no information about how people in some cultures perceive animals, and, at the same time, the apparent lack of research interest in certain regions might stem from those very cultural differences that shape social perception.

Finally, there is a need for studies addressing how people’s perceptions may change after visiting a zoo or aquarium. This necessity would require comparing results before and after the visit, and our review found almost no pre–post studies. Even if this may be related to the high cost of this kind of research (as compared to single-point studies), these works seem increasingly relevant in the context of growing societal concern about animals, how we treat them, and the role that zoos and aquariums play in that animal care and in promoting the wellbeing of all of them.

## Figures and Tables

**Table 1:** Types of data extracted from the selected papers.

Types	Data	Variables
Bibliometric characteristics	Year of publication	1993–2023
	Journal	Name of the journal
	Authors’ names ^1^	
	Authors’ gender ^2^	Female; male; other
	Authors’ affiliations	
	Authors’ country	As stated in the author’s affiliation/s
	Study keywords	Name of the journal
Methodology	Date of study	Start and end dates
	Tools	Questionnaire; interview; observation; other
	Questionnaire items	Open-answer; closed-answer; Likert scale; not disclosed
	Moment of application	Pre-visit; during the visit; post-visit; follow-up; does not apply (for studies in non-visitors); unspecified/unclear
Sample	Participating zoo/aquarium	Name of the institutions where the study was carried out
	Country of zoo/aquarium	As stated in the paper text
	Target group	Visitors; staff; general public AND adult; family; children
	Number of participants	
Main results		

^1^ Authors of more than one paper were counted as many times as they appeared. ^2^ We inferred the authors’ gender from their signature names and the biographical data in their institutions or academic repositories.

## Data Availability

The original contributions presented in this study are included in the Supplementary Materials. Further inquiries can be directed to the corresponding author.

## Supplementary Materials

The following supporting information can be downloaded at https://www.mdpi.com/article/10.3390/ani14243671/s1, Table S1: List of reviewed publications with the tools used, the questionnaire items, and the point in time when the data were collected.
